# Switching salts to protect against cardiovascular disease

**DOI:** 10.1038/s43856-021-00040-0

**Published:** 2021-10-18

**Authors:** Ben Abbott

**Affiliations:** Communications Medicine, https://www.nature.com/commsmed

## Abstract

High levels of salt in the diet have been associated with high blood pressure and poor cardiovascular health. A recent trial in *The New England Journal of Medicine* investigates whether a salt substitute could decrease the rate of strokes, other cardiovascular events and deaths in a high risk population.

**Figure:**
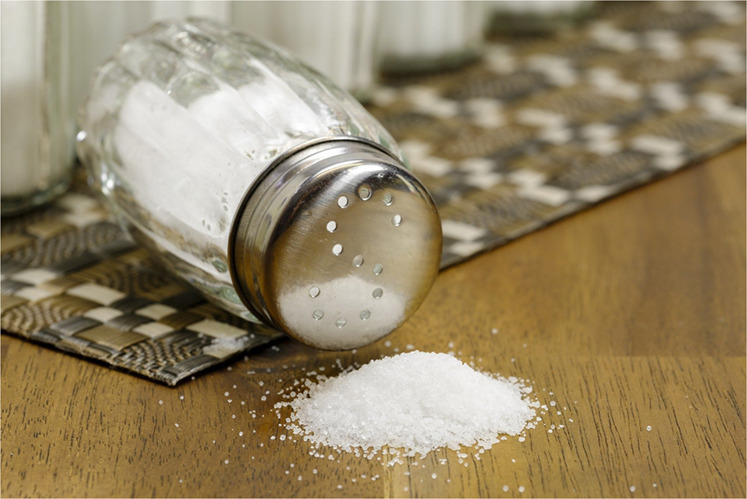
Pixabay

While the impact of salt on our health and the appropriate level of salt in our diet have long been debated, a number of studies have linked high dietary sodium intake with high blood pressure and increased risk of cardiovascular disease, stroke and death. Low dietary potassium intake has been associated with similar effects.

Substituting some of the sodium chloride in salt for potassium chloride may mitigate some of its harmful cardiovascular effects, with studies showing that salt substitutes with reduced sodium and added potassium can lower blood pressure. However, large-scale long-term randomised controlled trials of the effects of salt substitutes on cardiovascular outcome and mortality, performed in high-risk adults within the community, have been lacking.

The findings of the Salt Substitute and Stroke Study (SSaSS) were recently published in *The New England Journal of Medicine* by Neal and colleagues^1^. This open-label, cluster-randomised trial recruited over 20,000 people in 600 rural villages across China. Participants were adults with a history of stroke or aged over 60 with poorly controlled blood pressure. Villages were randomised to either regular salt or a salt substitute comprised of 75% sodium chloride and 25% potassium chloride by mass, which they were asked to use in all household cooking and food preservation.

Over a mean follow-up of 4.74 years, the authors observed a significant reduction in the rate of strokes—the primary outcome of the study—in the intervention group. Secondary outcomes of major cardiovascular events (including non-fatal stroke, non-fatal acute coronary syndrome and death from any vascular cause) and death from any cause were also significantly reduced. No significant increase was observed in the rate of clinical hyperkalaemia, which is a higher than normal potassium level in the blood, and was the study’s safety outcome.

It remains to be established whether data from SSaSS, carried out in rural China, can be generalised to populations in other geographical regions. However, this landmark study is the first large and robust trial to show a clear benefit for salt substitution in reducing the risk of adverse cardiovascular outcomes. These findings could not only pave the way towards implementing this type of dietary intervention in high risk populations, but also help to settle the wider debate on the role of salt substitutes in cardiovascular disease prevention.
